# Reward Sensitivity at Age 13 Predicts the Future Course of Psychopathology Symptoms

**DOI:** 10.3389/fpsyt.2022.818047

**Published:** 2022-03-11

**Authors:** Raniere Dener Cardoso Melo, Robin N. Groen, Catharina A. Hartman

**Affiliations:** Interdisciplinary Center Psychopathology and Emotion Regulation, Department of Psychiatry, University Medical Center Groningen, University of Groningen, Groningen, Netherlands

**Keywords:** reward, Behavioral Activation System (BAS), transdiagnostic, psychopathology, longitudinal studies, development, adolescence

## Abstract

**Background:**

There are numerous observations of reward sensitivity being associated with different psychiatric disorders. Nonetheless, most studies investigating this relationship have been cross-sectional. Additionally, current knowledge is fragmentary as studies often investigate only one disorder at a time. The present study addresses these gaps by investigating whether reward sensitivity at age 13 predicts the course of nine psychopathology domains (attention and hyperactivity, autism spectrum, reactive aggression, proactive aggression, mood, anxiety, smoking, alcohol use, and cannabis use) over a 14-year follow-up period.

**Methods:**

We used dimensional outcomes on 2,523 individuals over five measurement waves between ages 13 and 26 of the Dutch Tracking Adolescents' Individual Lives Survey (TRAILS). Reward sensitivity was measured with the Behavioral Activation System (BAS) scale. The longitudinal associations between reward sensitivity and psychopathology were examined using growth curve analysis within a multilevel framework.

**Results:**

Reward sensitivity at age 13 was associated with changes in psychopathology over time. Reward sensitivity had a stable main effect on the future course of reactive and proactive aggression problems and anxiety problems. The effect of reward sensitivity increased over time for alcohol and cannabis use. *Post-hoc* analyses showed that reward sensitivity also had a stable effect on attention problems and hyperactivity and smoking when based on the fun-seeking subscale for both domains and when changing the informant who reported on attention problems and hyperactivity. No evidence was found for a longitudinal association between reward sensitivity and autism spectrum problems and mood problems.

**Conclusion:**

The current study provides evidence for the long-lasting effects of reward sensitivity on the course of different domains of psychopathology.

## Introduction

Rewards are essential for human behavior because they guarantee our survival (e.g., by eating and drinking water) and influence our positive emotional experiences, motivation, and learning processes (1). Nonetheless, people with high or low reward sensitivity might be more vulnerable to developing psychiatric disorders. This is based on numerous observations that individual differences in reward sensitivity are associated with a wide range of psychiatric disorders. Examples include attention deficit and hyperactivity disorder (ADHD), autism spectrum disorder (ASD), disruptive behavior disorders (DBD), major depressive disorder (MDD), anxiety disorders, and substance use disorders (SUD) (2–9). Still, it is unclear whether extreme levels of reward sensitivity are merely part of the symptoms of psychopathology or play a role in the onset and course of psychiatric disorders.

One way of conceptualizing individual differences in reward sensitivity is based on Gray's reinforcement sensitivity model of personality (10). Gray proposes brain-behavioral systems that control behavioral activity. One of these systems is the Behavioral Activation System (BAS), which regulates approach motivation to attain rewards (10). The BAS has been largely used as a framework for understanding individual differences in reward sensitivity that are associated with psychopathology as measured by questionnaires. In this paper, we will use this framework and use reward sensitivity and BAS as interchangeable terms. The most common conceptualizations divide the BAS into three aspects/subscales that are all related to reward approach behavior but differ in what motivates the approach. Responsiveness relates to approach behavior that is motivated by the reward. Drive relates to the perseverance in pursuing a reward once selected. The last aspect, fun-seeking, relates to the motivation to seek new and intense rewards. Prior research showed some evidence for high reward sensitivity in ADHD, DBD, SUD, and anxiety disorders (2–5, 9). In contrast, MDD has been associated with low reward sensitivity (6). Even though research on reward sensitivity measured by questionnaires in ASD is scarce, some studies have described low reward sensitivity in ASD (7, 8).

Although extreme levels of reward sensitivity have been associated with many different psychiatric disorders, studies often investigated only one disorder at a time. As a result, methodological differences across these studies (e.g., design, sample characteristics, statistical approach) make our current knowledge fragmentary. Careful mapping of reward sensitivity to various psychiatric disorders might aid in filling this knowledge gap, elucidating shared and specific alterations associated with the onset and course of these disorders. Additionally, it is important to note that most research on the relationship between reward sensitivity and psychopathology has been cross-sectional, meaning that reward sensitivity and psychopathology were assessed during the same occasion. Therefore, it is not possible to draw any conclusions on a potential mechanistic role of extreme levels of reward sensitivity in psychopathology. One first step to further the field is to study the potential predictive role of reward sensitivity on the course of psychopathology over time.

Considering such a developmental perspective is also relevant because many psychiatric disorders have an onset in youth and are often chronic (11). Among all life phases, adolescence and the transition into adulthood stand out as an important period to study changes in psychopathology. Adolescence is a sensitive period of development that involves significant physical, sexual, cognitive, social, and emotional changes. Many psychiatric disorders have their onset during this period, such as MDD, anxiety disorders, and SUD. Other disorders may or may not remit, such as ADHD or DBD, or potentially slightly improve, such as ASD. Thus, in this period of considerable individual differences in change, symptoms of any of these disorders may improve or exacerbate into adulthood as a function of reward sensitivity.

This study investigated whether reward sensitivity at age 13 predicted the course of different psychopathology domains longitudinally from age 13 to 26. We focused on the following nine domains of psychopathology: attention and hyperactivity, autism spectrum, reactive aggression, proactive aggression, mood, anxiety, smoking, alcohol use, and cannabis use. Although most research on the relationship between reward sensitivity and psychopathology has focused on discrete categorical psychiatric diagnosis, we used dimensional measures that are closer to the observed continuous nature of severity of psychopathology symptoms and are therefore optimal to study developmental change.

Based on the current literature, we hypothesized that reward sensitivity would significantly predict the course of the psychopathology domains included in the study. Specifically, we expected (1) a positive association between reward sensitivity and the course of attention problems and hyperactivity, reactive and proactive aggression problems, anxiety problems^1^, and substance use problems (i.e., smoking, alcohol use, and cannabis use) in two forms:

a) Less improvement over time in individuals with higher levels of reward sensitivity in problem domains with a normative decreasing course (attention problems and hyperactivity, and aggression problems).
b) More worsening over time in individuals with higher levels of reward sensitivity in problem domains with a normative increasing course (anxiety and substance use problems).

By contrast, we expected (2) a negative association between reward sensitivity and the course of autism spectrum and mood problems. This negative association may take two forms:

a) More improvement over time in those with higher levels of reward sensitivity in problem domains with a (slightly) normative decreasing course (autism spectrum problems).
b) Less worsening over time in those with higher levels of reward sensitivity in problem domains with a normative increasing course (mood problems).

## Materials and Methods

This study's aims, hypotheses, and analyses were preregistered on the Open Science Framework (https://osf.io/47qwk).

### Sample and Design

We used data from the Tracking Adolescents' Individual Lives Survey (TRAILS). TRAILS is a Dutch longitudinal study designed to track development from preadolescence into adulthood, starting at 11 years old. Adolescents were recruited from primary schools of five municipalities in the North of the Netherlands. TRAILS comprises a general population cohort (*N* = 2,230) and a clinical cohort (TRAILS-CC; *N* = 543). TRAILS-CC was designed to selectively sample individuals at heightened risk for mental illness within the TRAILS cohort study. This clinical cohort consists of individuals referred to child psychiatric outpatient clinics before 11 years old. TRAILS has relatively high retention rates, ranging between 73 and 96% for the general population cohort and 73 and 85% for TRAILS-CC. An extensive description of the sampling procedures for TRAILS has been published elsewhere (12). The ethics committee of the University Medical Center Groningen approved TRAILS, and informed consent was obtained from parents and subsequently from the adolescents for the different measurement waves.

From age 11 onwards, participants were assessed every two to three years. At the second measurement wave (T2; 13 years old), reward sensitivity was assessed, which we use as the starting point in this study. Therefore, we used data from five measurement waves (T2–T6). From the baseline measurement (T1), we only used information on sex, parental socioeconomic status (SES), and intelligence quotient (IQ) as covariates in the analyses. We selected all participants for whom data on reward sensitivity at T2 was available. This amounted to 2,523 participants in the study at age 13, of which 48.3% were female. Although attrition is relatively low in TRAILS, data was imputed to maximize the number of complete cases per scale (see Supplementary Data Sheet S2). After imputation, over 90% of the cases were complete at baseline (T2). Additionally, we imputed data for attention problems and hyperactivity, reactive aggression problems, and autism spectrum problems at specific waves. Details are given below in the measurements section. Participants were ~13 years at baseline (T2), 16 years at T3, 19 years at T4, 22 years at T5, and 26 years at T6 [*M*_ageT2_ (SD) = 13.44 (0.61); *M*_ageT3_ (SD) = 16.20 (0.71); *M*_ageT4_ (SD) = 19.07 (0.62); *M*_ageT5_ (SD) = 22.22 (0.67); *M*_ageT6_ (SD) = 25.72 (0.64)]. Most participants had an average SES (50.2%), and smaller groups had low (24.0%) and high SES (25.8%). Mean (SD) IQ at baseline was 95.57 (15.02).

### Measurements

#### Behavioral Activation Scale (BAS)

Reward sensitivity was assessed at age 13 (T2) with the parent-rated BAS scale from the Behavioral Inhibition and Activation Scales (BIS/BAS Scales) (13). The questionnaire consists of 13 items divided into three subscales (i.e., responsiveness, drive, and fun-seeking). The total BAS scale was calculated as the mean score of the three BAS subscales. Items were scored on a 4-point scale, coded as 1–4 (1 “very untrue”; 4 “very true”). Internal consistency of the total scale was satisfactory (α = 0.75). In *post-hoc* exploratory analyses, we used the subscales with α = 0.64, α = 0.65, α = 0.44 for, respectively, responsiveness, drive, and fun-seeking.

#### Child/Adult Behavior Checklist

Attention problems and hyperactivity and reactive aggression problems were assessed with the parent-rated Child and the Adult Behavior Checklists (CBCL; ABCL) (14, 15) at waves T2, T3, and T5. Data were imputed at T4 and T6 (see Supplementary Data Sheet S2). The scale measuring attention problems and hyperactivity consists of seven items in the CBCL and 13 items in the ABCL, while the one measuring reactive aggression problems consists of 18 items in the CBCL and 16 items in the ABCL. We measured them as mean scores, and items were scored on a 3-point scale, coded as 0–2 (0 “not at all”; 2 “clearly or often”). Internal consistency was satisfactory across waves for both attention problems and hyperactivity (α between 0.82 and 0.88) and reactive aggression problems (α between 0.76 and 0.88). In sensitivity analyses, we used the overlapping items between childhood and adult versions which were 6 items for attention problems and hyperactivity (α between 0.74 and 0.80), and 16 items for reactive aggression problems (α between 0.84 and 0.90).

#### Youth/Adult Self Report

Mood and anxiety problems were assessed with the self-rated Youth and Adult Self Report (YSR; ASR) (14, 15) at T2, T3, T4, T5, and T6. The scale measuring mood problems consists of 13 items in the YSR and 14 items in the ASR, and the one measuring anxiety problems consists of six items in the YSR and seven items in the ASR. We measured them as mean scores, and items were scored on a 3-point scale, coded as 0–2 (0 “not at all”; 2 “clearly or often”). Internal consistency was satisfactory across waves for both mood (α between 0.76 and 0.87) and anxiety problems (α between 0.63 and 0.80). In sensitivity analyses, we used the overlapping items between childhood and adult versions which were 12 items for mood problems (α between 0.74 and 0.85), and five items for anxiety problems (α between 0.63 and 0.79).

#### Child/Adult Social Behavior Questionnaire

Autism spectrum problems were assessed with the parent-rated Child and the Adult Social Behavior Questionnaires (CSBQ; ASBQ) (16, 17) at waves T2, T3, T4, and T6. Data were imputed at T5 (see Supplementary Data Sheet S2). We used the four comparable subscales across instrument versions (i.e., reduced contact, reduced social insight, resistance to changes, and stereotyped behavior). In total, 30 items were used from the CSBQ and 28 items from the ASBQ, and we measured these problems as a mean score of all items. Items were scored on a 3-point scale, coded as 0–2 (0 “not at all”; 2 “clearly or often”). Internal consistency was satisfactory across waves (α between 0.92 and 0.93). In sensitivity analyses, we used the 20 overlapping items between childhood and adult versions (α between 0.88 and 0.89).

#### Antisocial Behavior Questionnaire

Proactive aggression problems were assessed with the self-rated Antisocial Behavior Questionnaire (ASBQ) (18) at T2, T3, T4, T5, and T6. The ASBQ included a slightly different number of items at each wave: 26, 28, 29, 29, and 26 items at waves T2, T3, T4, T5, and T6, respectively. We measured these problems as a mean score, and items were scored on a 5-point scale, coded as 0–4 (0 “no/never”; 4 “seven times or more”). Internal consistency was satisfactory across waves (α between 0.69 and 0.86). In sensitivity analyses, we used the 18 overlapping items between all versions (α between 0.69 and 0.84).”

#### Self-Rated Substance Use Problems

Smoking, alcohol use, and cannabis use were assessed through a self-rating survey at T2, T3, T4, T5, and T6. For smoking, we measured the self-rated frequency of cigarette smoking in the past month on a 7-point scale (coded as 0 “no cigarettes” /6 “more than 20 cigarettes”). We calculated a mean score for alcohol use, reflecting the average number of alcoholic beverages consumed during a regular day that could range from zero to 20 beverages. For cannabis use, we measured the self-rated frequency of monthly cannabis use that could go from zero to 40 times. Further details are provided in Supplementary Data Sheet S3.

#### Covariates

Baseline psychopathology, baseline age, IQ, parental SES, and sex were used to adjust for potential confounding. These variables are known to be linked to developmental course of psychopathology and could potentially be linked to reward sensitivity as well (19–22). Adjustment for these covariates thus aids the interpretation of the associations that we may find between reward sensitivity and psychopathology. Baseline psychopathology was computed as the score for each psychopathology domain at T2. Baseline age was computed as the age of each participant at T2. IQ was assessed at T1 with the shortened version of the Wechsler Intelligence Scale for Children-Revised (WISC-R) (23). IQ was estimated for each person using the Vocabulary and Block Design subtests of the WISC-R (23–25). Parental socioeconomic status (SES) was based on a combined score of five Z-transformed indicators, i.e., educational attainment (both parents), profession (both parents) and household income. Next, we split parental SES into three categories (lowest 25% “low,” middle 50% “average,” highest 25% “high”), but only for descriptive purposes (i.e., SES was a continuous variable in the growth curve analyses). Finally, sex was assessed at T1 and coded as a binary variable (0 “female” /1 “male”).

Please note that we used different informants for different problem domains. This distinction is based on previous literature that has shown that parents are generally better at reporting on ADHD and externalizing behavior problems, but less valid reporters of internalizing behavior problems (26–28). The only exception was proactive aggression problems, for which we used self-reported data. This was done because the ASBQ uses only self-report of proactive antisocial behaviors.

### Statistical Analyses

We calculated, first, cross-sectional correlations of the total BAS scale and subscales with psychopathology at baseline^2^. These allow for comparison with the (mostly) cross-sectional literature and exploration of possible stronger associations with psychopathology at the subscale level compared to the total scale level. Second, for descriptive purposes, we provided the mean scores on the nine domains of psychopathology at T2, T3, T4, T5, and T6, illustrating the overall developmental change over time.

Next, we modeled the course of psychopathology over time, using growth curve analyses within a multilevel framework. This was done for each psychopathology domain separately. Psychopathology and time were person-centered at baseline. For the time variable, this meant that the age of each participant at baseline was subtracted from the ages at T2, T3, T4, T5, and T6, thus representing the follow-up time in the study. For psychopathology, this meant that the starting scores of each participant on the nine psychopathology domains were subtracted from the scores at T2, T3, T4, T5, and T6, as such representing the course relative to T2 (i.e., an intercept of zero at T2). This approach allows baseline psychopathology to be added as a covariate, thus disentangling the starting level of psychopathology from the effect of reward sensitivity on the *change* of psychopathology, which is the focus of our study (29). First, we calculated the intraclass correlation coefficients (ICC) from the unconditional means models. Next, the growth curve was modeled with a fixed intercept representing the starting point (T2) and a random time effect representing the course over time (T2–T6). The total BAS scale was subsequently added as main effect, indicating the association with the rate of change of the outcome, and in interaction with time, indicating the stability of the association over time. We adjusted for the level of psychopathology at baseline and in interaction with time (e.g., when modeling the course of mood problems, we adjusted for mood problems at baseline and in interaction with time) (29). Baseline age, IQ, and SES (all mean-centered), and sex were additional covariates. Finally, we rescaled all outcome variables to a 0–100 points scale for comparability across the different findings when visualizing the results. Given that this is a linear transformation, rescaling is fully compatible with the standardized regression coefficients.

### Sensitivity Analyses

Three sensitivity analyses were performed. First, our main analysis used the original scales from the different measurement instruments. However, these scales include different items over time because different developmental periods require different behaviors. For example, “being expelled from class” is only appropriate when schooling is relevant for everyone; after that, this item is no longer included, but other items become relevant, such as “misinforming tax authorities” and “selling drugs.” Therefore, our first sensitivity analyses involved re-running our models to check whether our results were influenced by these different developmentally appropriate items at different waves using only the fully overlapping items across the waves. We did this for attention problems and hyperactivity, autism spectrum problems, proactive and reactive aggression problems, mood problems, and anxiety problems. For substance use, all items used were already comparable across the waves. A second sensitivity analysis pertained specifically to the reactive and proactive aggression problems scales. These came from different instruments and had some items with similar content. Therefore, we re-ran the two models without the overlapping items to test whether overlapping items explained potential overlap in findings for these two domains. Finally, we re-ran our models adjusting for psychotropic medication use over time^3^. This was done to check whether our results were influenced by medication use.

## Results

### Descriptive Statistics

The total BAS mean score (SD) was 2.91 (0.41). BAS subscales responsiveness, drive, and fun-seeking had mean scores (SD) of 3.22 (0.49), 2.74 (0.61), 2.70 (0.50), respectively. Table 1 shows the cross-sectional associations between the total BAS and subscale mean scores and psychopathology at baseline. Specific BAS subscales had a somewhat higher cross-sectional correlation with some psychopathology domains than the total BAS score used in our main analyses. That is, compared to the overall mean score, attention problems and hyperactivity, proactive and reactive aggression problems, and substance use problems were somewhat more strongly correlated with drive and fun-seeking, while for anxiety, this was the case with responsiveness. On the other hand, autism spectrum problems were slightly more strongly correlated with drive. Although relatively small differences, these findings prompted additional exploratory *post-hoc* analyses to determine if targeted subscale analyses based on these correlational patterns would alter our main conclusions, as described in section *Post-hoc* Exploratory Analyses below.

**Table 1:** Correlations between total BAS and subscale mean scores and psychopathology domain scores at baseline.

	**Attention problems and hyperactivity**	**Autism spectrum problems**	**Proactive aggression problems**	**Reactive aggression problems**	**Mood problems**	**Anxiety problems**	**Alcohol use**	**Smoking**	**Cannabis use**
Total BAS	0.13^**^	0.05^**^	0.14^**^	0.15^**^	0.11^**^	0.11^**^	0.04^*^	0.04^*^	0.04
Responsiveness	0.02	0.03	−0.01	0.04	0.10^**^	0.17^**^	−0.05^**^	−0.02	−0.03
Drive	0.13^**^	0.08^**^	0.16^**^	0.17^**^	0.07^**^	0.03	0.08^**^	0.05^**^	0.06^**^
Fun-seeking	0.17^**^	0.04	0.17^**^	0.15^**^	0.10^**^	0.06^**^	0.08^**^	0.10^**^	0.08^**^

* *The bold values indicates that are statistically significant. The symbol indicates the value p < 0.05 and*

** *symbol indicates the values p < 0.001*.

Table 2 presents the mean scores on the nine psychopathology domains at T2, T3, T4, T5, and T6. These normative developmental patterns are in line with expectation, i.e., increasing over time for mood problems, anxiety problems, smoking, alcohol use, and cannabis use, decreasing over time for attention problems and hyperactivity, reactive aggression problems, and proactive aggression problems, and remaining stable (with only a slight decrease) for autism spectrum problems.

**Table 2:** Mean scores for psychopathology domains over time.

	**T2**	**T3**	**T4**	**T5**	**T6**
Attention problems and hyperactivity	0.52 (0.47)	0.47 (0.45)	0.44 (0.33)	0.37 (0.36)	0.32 (0.30)
Autism spectrum problems	0.23 (0.26)	0.23 (0.26)	0.21 (0.25)	0.22 (0.23)	0.21 (0.26)
Reactive aggression problems	0.29 (0.30)	0.26 (0.30)	0.25 (0.21)	0.19 (0.25)	0.19 (0.21)
Proactive aggression problems	0.28 (0.32)	0.23 (0.30)	0.08 (0.18)	0.06 (0.13)	0.05 (0.11)
Mood problems	0.28 (0.26)	0.30 (0.27)	0.31 (0.31)	0.33 (0.32)	0.40 (0.36)
Anxiety problems	0.37 (0.32)	0.34 (0.31)	0.40 (0.36)	0.42 (0.38)	0.52 (0.42)
Smoking	0.27 (1.04)	1.01 (1.84)	1.40 (2.04)	1.48 (2.05)	1.20 (1.89)
Alcohol use	0.18 (0.43)	0.89 (1.26)	1.37 (1.57)	1.39 (1.55)	1.33 (1.66)
Cannabis use	0.11 (1.26)	1.08 (5.07)	1.82 (6.54)	1.85 (6.59)	2.20 (8.53)

*Mean scores are presented in their original scales. Attention problems and hyperactivity, autism spectrum problems, reactive aggression problems, mood problems, and anxiety problems are on a 3-point scale. Proactive aggression problems are on a 5-point scale. Smoking is on a 7-point scale. Alcohol use is on a 21-point scale. Cannabis use is on a 41-point scale*.

### Intraclass Correlations

We first calculated the intraclass correlations (ICC). An ICC of at least 10% is considered appropriate to account for clustering effects over time with a multilevel model (30). The ICC was 0.47 for attention problems and hyperactivity, 0.41 for autism spectrum problems, 0.47 for reactive aggression problems, 0.43 for proactive aggression problems, 0.34 for mood problems, 0.39 for anxiety problems, 0.35 for smoking, 0.20 for alcohol use, and 0.27 for cannabis use. Therefore, 20 to 47% of the variance in the psychopathology domains was attributable to clustering. The intraclass correlations thus indicate both stability of psychopathology and change from early adolescence to young adulthood.

### Predictive Role of Reward Sensitivity on the Course of Psychopathology

We then tested whether reward sensitivity predicted the course of psychopathology while controlling for the level of baseline psychopathology, age at baseline, sex, SES, and IQ. Model estimates are provided in Table 3.

**Table 3:** Multilevel growth curve model estimates for all psychopathology domains.

	**Attention and hyperactivity**	**Autism spectrum**	**Reactive aggression**	**Proactive aggression**	**Mood**	**Anxiety**	**Smoking**	**Alcohol**	**Cannabis**
	**Est (SE)**	**Est (SE)**	**Est (SE)**	**Est (SE)**	**Est (SE)**	**Est (SE)**	**Est (SE)**	**Est (SE)**	**Est (SE)**
**Fixed Effects**									
(Intercept)	−0.16 (0.18)	−0.06 (0.13)	0.14 (0.13)	−0.77 (0.08)^**^	0.72 (0.19)^**^	−0.04 (0.22)	4.66 (0.40)^**^	0.49 (0.10)^**^	−0.11 (0.21)
Follow-up time	−0.75 (0.03)^**^	−0.04 (0.02)	−0.38 (0.02)^**^	−0.54 (0.01)^**^	0.39 (0.04)^**^	0.62 (0.05)^**^	1.95 (0.09)^**^	0.56 (0.02)^**^	0.54 (0.05)^**^
Reward sensitivity	0.24 (0.13)	0.07 (0.10)	0.22 (0.10)^*^	0.20 (0.07)^*^	0.23 (0.15)	0.38 (0.16)^*^	0.04 (0.30)	−0.01 (0.08)	0.14 (0.15)
Reward sensitivity^*^Time	0.03 (0.03)	0.02 (0.02)	0.01 (0.02)	−0.00 (0.01)	0.01 (0.04)	0.00 (0.05)	0.18 (0.09)	0.06 (0.02)^*^	0.09 (0.05)^*^
Baseline psychopathology	−2.29 (0.14)^**^	−1.18 (0.10)^**^	−1.54 (0.10)^**^	−1.46 (0.07)^**^	−2.13 (0.15)^**^	−3.34 (0.16)^**^	−1.17 (0.31)^**^	0.00 (0.08)	−0.44 (0.17)^*^
Baseline psychopathology ^*^Time	−1.00 (0.03)^**^	−0.39 (0.02)^**^	−0.64 (0.02)^**^	−0.64 (0.01)^**^	−0.60 (0.04)^**^	−0.79 (0.05)^**^	−0.96 (0.10)^**^	−0.14 (0.02)^**^	−0.19 (0.06)^*^
Baseline age	−0.47 (0.12)^**^	−0.29 (0.09)^*^	−0.37 (0.08)^**^	−0.22 (0.04)^**^	0.07 (0.13)	0.07 (0.14)	−0.18 (0.27)	0.28 (0.07)^**^	0.36 (0.14)^*^
Sex	0.57 (0.23)^*^	0.23 (0.17)	−0.32 (0.17)	1.02 (0.09)^**^	−2.27 (0.26)^**^	−2.79 (0.30)^**^	−0.43 (0.54)	1.48 (0.14)^**^	1.36 (0.28)^**^
SES	−0.27 (0.12)^*^	−0.27 (0.09)^*^	−0.23 (0.09)^*^	−0.19 (0.05)^**^	−0.18 (0.14)	−0.12 (0.16)	−1.20 (0.29)^**^	0.14 (0.07)	0.11 (0.15)
IQ	−0.02 (0.13)	−0.24 (0.09)^*^	−0.21 (0.09)^*^	−0.19 (0.05)^**^	0.13 (0.14)	−0.47 (0.16)^*^	−0.93 (0.29)^*^	0.01 (0.07)	0.07 (0.15)
**Random effects**									
Variance	1.03	0.71	0.60	0.01	1.64	3.22	11.45	0.44	3.02
Residual variance	62.12	31.59	32.78	17.07	80.21	96.08	337.23	23.38	88.36
ICC	0.46	0.52	0.48	0.02	0.48	0.60	0.61	0.46	0.61

* *Est, estimate; SE, standard error. The bold values indicates that are statistically significant. The symbol indicates the value p < 0.05 and*

** *symbol indicates the values p < 0.001*.

**Figure 1:**
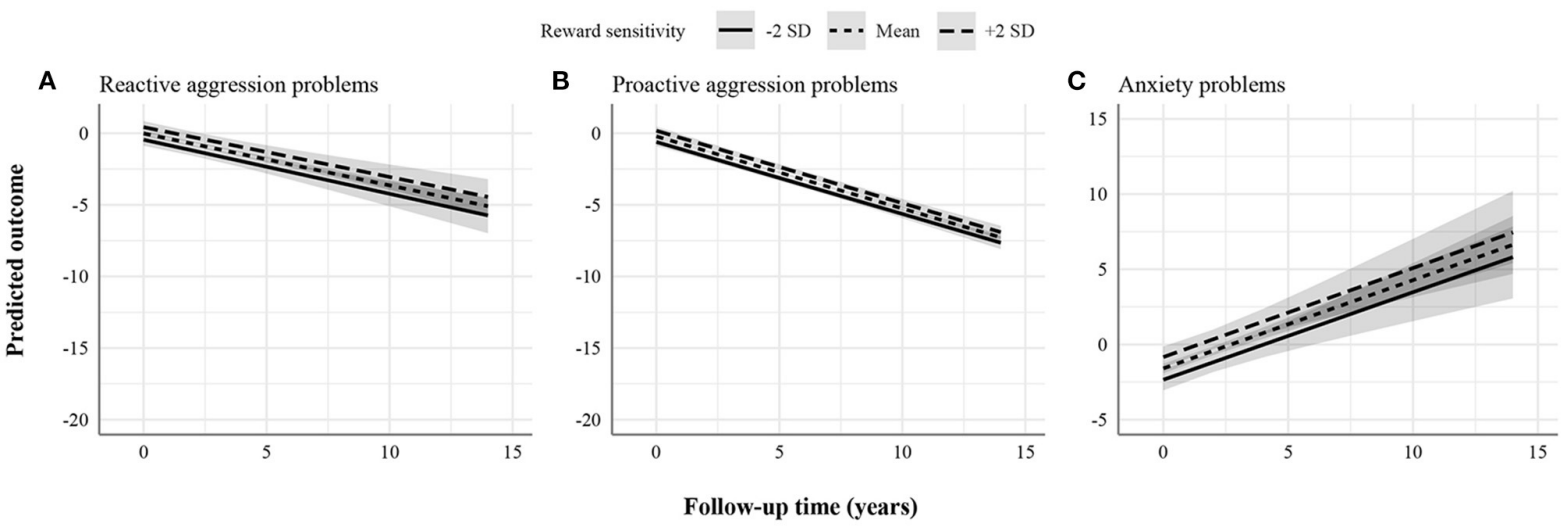
Statistically significant stable associations between reward sensitivity and psychopathology domains. **(A)** Reactive aggression problems. **(B)** Proactive aggression problems. **(C)** Anxiety problems. The figures display the effect of reward sensitivity on psychopathology based on its main effect and in interaction with time. Here, the x-axes show follow-up time in years, whereas the different lines show reward sensitivity levels at 2 SD above and below the mean. The y-axes represent the predicted outcome with all covariates at mean levels and sex coded as 0 (males). The y-axes are only partly shown for better visualization of the findings.

**Figure 2:**
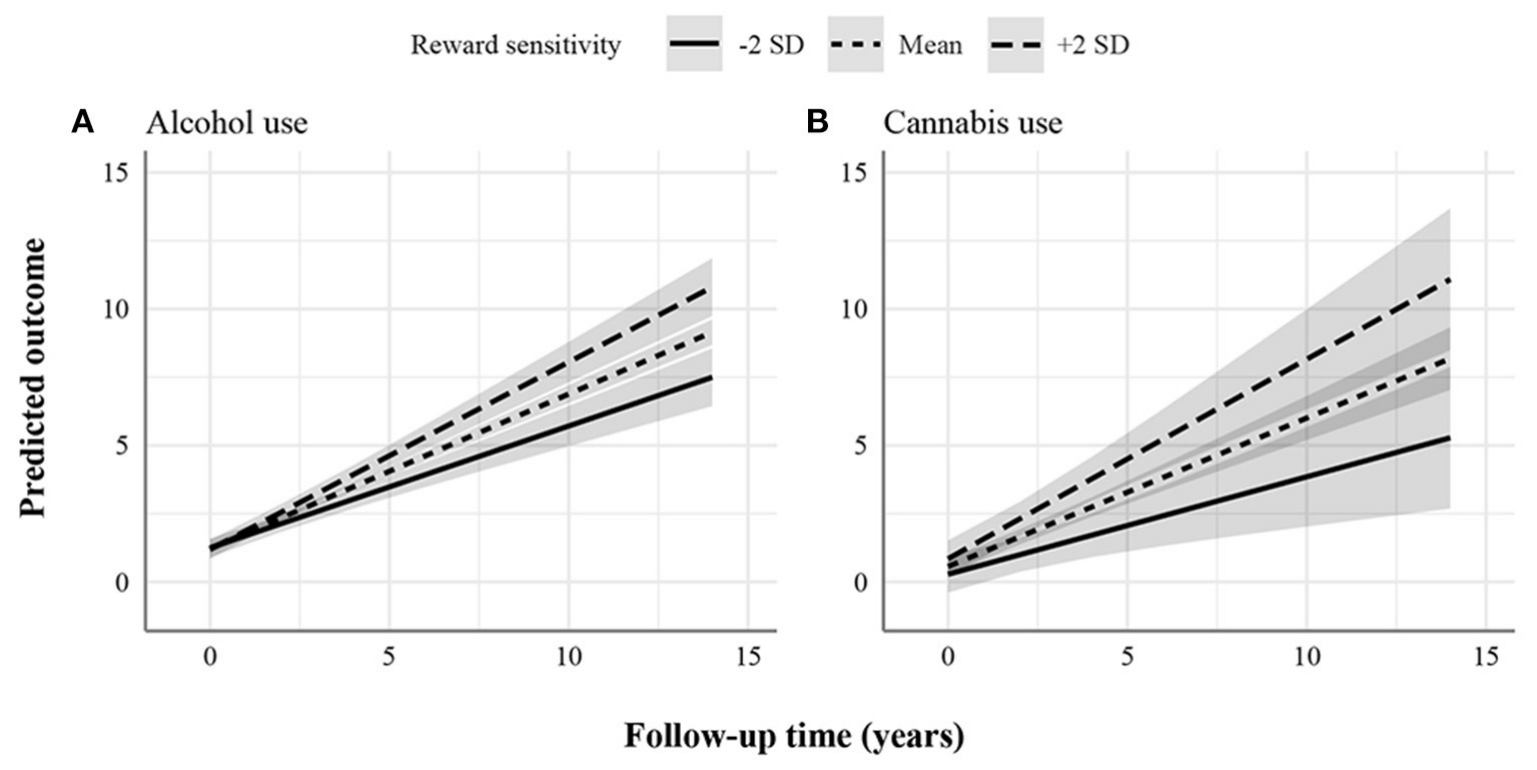
Statistically significant increasing associations between reward sensitivity and psychopathology domains. **(A)** Alcohol use. **(B)** Cannabis use. The figures display the effect of reward sensitivity on psychopathology based on its main effect and in interaction with time. Here, the x-axes show follow-up time in years, whereas the different lines show reward sensitivity levels at 2 SD above and below the mean. The y-axes represent the predicted outcome with all covariates at mean levels and sex coded as 0 (males). The y-axes are only partly shown for better visualization of the findings.

**Figure 3:**
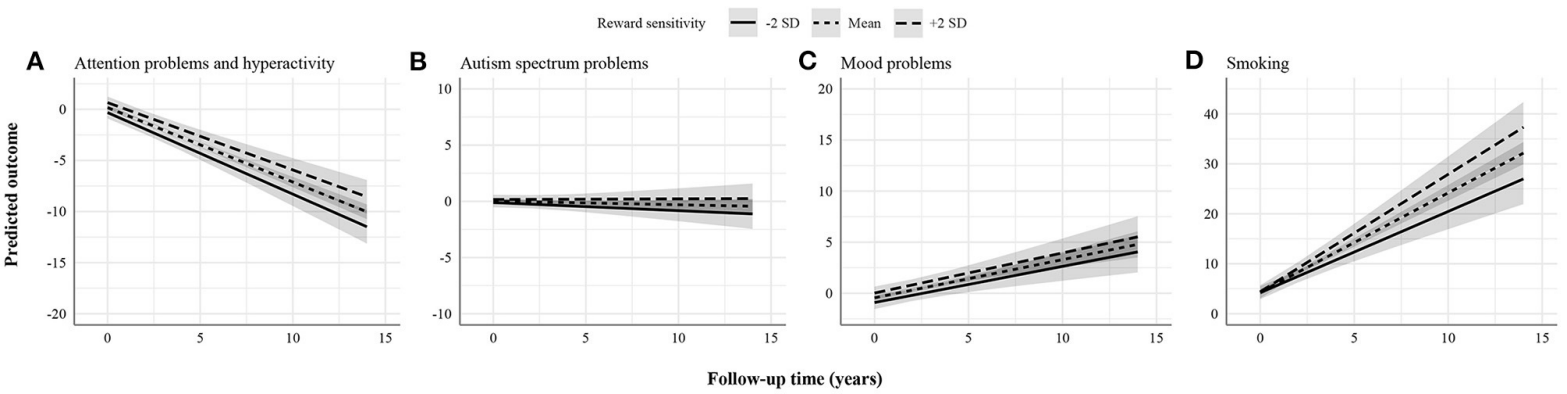
Statistically non-significant associations between reward sensitivity and psychopathology domains. **(A)** Attention problems and hyperactivity. **(B)** Autism spectrum problems. **(C)** Mood problems. **(D)** Smoking. The figures display the effect of reward sensitivity on psychopathology based on its main effect and in interaction with time. Here, the x-axes show follow-up time in years, whereas the different lines show reward sensitivity levels at 2 SD above and below the mean. The y-axes represent the predicted outcome with all covariates at mean levels and sex coded as 0 (males). The y-axes are only partly shown for better visualization of the findings.

Figures 1–3 display the predicted response based on the main effect of reward sensitivity and its effect in interaction with time, with all covariates at mean levels and sex coded as 0 (males). Note that different psychopathology domains change differently over time. For instance, proactive aggression problems decreased more strongly over time compared to reactive aggression problems (Figure 1). Thus, to be able to compare the effect of reward sensitivity across different psychopathology domains, the total change over time for each domain needs to be considered. To do that, we use percentages to express differences between the predicted change over time in psychopathology of individuals with low (i.e., 2 SD below the mean) and high (i.e., 2 SD above the mean) reward sensitivity. Percentage difference is the difference between the two values divided by the average of the two values shown as a percentage.

We found a positive effect of reward sensitivity on the course of reactive aggression, proactive aggression, and anxiety problems (Figure 1). This association was present in two forms depending on whether normative developmental patterns of psychopathology decrease or increase, on average. First, in reactive and proactive aggression problems, individuals with higher reward sensitivity showed less improvement over time than those scoring lower. The total change over the follow-up period predicted for individuals with low and high reward sensitivity in reactive aggression problems was 5.29 and 4.86 points, respectively. The percentage difference in total change over time between individuals with low and high reward sensitivity was 8.47%. For proactive aggression problems, the total change over the follow-up period predicted for individuals with low and high reward sensitivity was 7.04 and 6.73 points, respectively. The percentage difference in total change over time was 4.50%. Second, in anxiety problems, individuals with higher reward sensitivity showed more worsening over time than those scoring lower. The total change over the follow-up period predicted for individuals with low and high reward sensitivity in anxiety problems was 8.16 and 8.29 points, respectively. The percentage difference in total change over time was 1.58%. These effects were stable over time (i.e., interaction effects of reward sensitivity and time were not significant), meaning that the effect of reward sensitivity remained the same over the study period.

Additionally, we found a positive effect of reward sensitivity on the course of alcohol and cannabis use (Figure 2). This effect increased over time, as indicated by the significant interaction effect. Individuals with higher reward sensitivity showed more worsening (i.e., increasingly used more alcohol and cannabis) over time than those with lower levels. For alcohol use, the total change over the follow-up period predicted for individuals with low and high reward sensitivity was 6.25 and 9.61 points, respectively. The percentage difference in total change over time was 42%. For cannabis use, the total change over the follow-up period predicted for individuals with low and high reward sensitivity was 5.01 and 10.23 points, respectively. The percentage difference in total change over time was 68%.

Finally, there was no evidence found for the association between reward sensitivity and the course of attention problems and hyperactivity, autism spectrum problems, mood problems, and smoking (Figure 3). Particularly, findings for attention problems and hyperactivity were unexpected. These were further explored in *post-hoc* analyses by changing the informant (i.e., self-rated instead of parent-rated attention problems and hyperactivity).

### Sensitivity Analyses

Sensitivity analyses (i.e., [a] using the overlapping items across the waves instead of the original scales, [b] removing the overlapping items between reactive and proactive aggression, and [c] adjusting for psychotropic medication use) yielded highly similar results as our main analyses and conclusions remained unaltered. Model estimates are provided in Supplementary Tables S1, S2.

### Post-hoc Exploratory Analyses

First, based on the findings shown in Table 1, we checked if analyses based on the highest correlated BAS subscale would yield similar results to our main findings based on the total BAS score. Model estimates when considering specific subscales are provided in Supplementary Table S3. We observed similar or smaller effect sizes for proactive aggression problems, reactive aggression problems, autism spectrum problems, mood problems, anxiety problems, and alcohol use, indicating that the broad BAS mean score measure fits these outcomes (Supplementary Figure S1). On the other hand, we observed a positive effect of the fun-seeking subscale on the course of attention problems and hyperactivity (main effect: 0.36 vs. 0.22) and smoking (main effect: 0.90 vs. 0.06; interaction effect: 0.22 vs. 0.17) (Supplementary Figure S2). In addition, we observed a bigger effect size for cannabis use with a statistically significant main effect instead of a significant interaction effect (main effect: 0.47 vs. 0.14).

Second, no evidence was found for the association between reward sensitivity and the course of attention problems and hyperactivity in our main analysis. Since this was unexpected and given that we also had self-reported information on attention problems and hyperactivity, we decided to check whether discrepancies across different informants played a role. When using self-rated attention problems and hyperactivity, we found evidence for the association between reward sensitivity and attention problems and hyperactivity. Individuals with higher reward sensitivity showed persistently less improvement over time than those with lower (Supplementary Figure S3). The total change over the follow-up period predicted for individuals with low and high reward sensitivity in attention problems and hyperactivity was 17.65 and 16.17 points, respectively. The percentage difference in total change over time between individuals with low and high reward sensitivity was 8.75%.

## Discussion

This study investigated the predictive role of reward sensitivity on the course of psychopathology. We showed that reward sensitivity measured at age 13 was associated with changes over time in reactive and proactive aggression problems, anxiety problems, and alcohol and cannabis use. High reward sensitivity was associated with less decline at each time point in reactive and proactive aggression problems, with a higher increase in anxiety problems at each time point, and with a higher increase in alcohol and cannabis use over time. While the effects were stable over time for reactive and proactive aggression problems and anxiety problems, the effect of reward sensitivity increased over time for alcohol and cannabis use. *Post-hoc* analyses additionally revealed a stable effect for attention problems and hyperactivity when using self-rated measures of psychopathology and when considering specific BAS subscales. Likewise, for smoking, both stable and increasing effects were observed when considering specific BAS subscales. These results show that high reward sensitivity is shared across multiple psychopathology domains, although no role in mood and autism spectrum problems was observed.

The findings that high reward sensitivity was associated with reactive and proactive aggression problems, anxiety problems, alcohol use, and cannabis use, and in the *post-hoc* analysis also with attention problems and hyperactivity, as well as smoking are in agreement with previous cross-sectional studies. For instance, previous research has shown evidence for a positive association between reward sensitivity and both types of aggression (3). Similarly, the associations between reward sensitivity and problems related to attention, hyperactivity, and substance use have been widely explored cross-sectionally (2, 4, 9). The findings on the association between reward sensitivity and anxiety are somewhat mixed. On the one hand, a recent meta-analysis has reported no evidence for this association when measuring reward sensitivity with questionnaires (6). On the other hand, Barker and colleagues have reviewed findings in clinical and cognitive neuroscience that support an increased reward sensitivity in anxiety (5). This positive association is expected due to the activation of both reward and punishment systems that typically happens in highly novel, ambiguous, and unpredictable contexts, resulting in an approach-avoidance conflict (31). We extend these previous findings by showing that these associations can also be observed longitudinally and that they are long-lasting. Additionally, for alcohol and cannabis use, and *post-hoc* specifically for fun-seeking predicting smoking, reward sensitivity effects became stronger with time. Our findings suggest, on the one hand, stable effects for childhood-onset psychiatric problems. On the other hand, we observed larger effects as they increase over time for adolescent- and young adult-onset problems, specifically substance use. During this normative increase, high reward sensitivity may accelerate the use of these substances.

Contrary to our hypotheses and prior research, we did not find that reward sensitivity was associated with less decline in attention problems and hyperactivity over the course of adolescence. Discrepancies across different informants seemed to play a role, i.e., we used a self-rated measure for reward sensitivity but a parent-rated measure for attention problems and hyperactivity in our main analyses. However, findings were as expected when using self-ratings of attention problems and hyperactivity in *post-hoc* analyses. Our findings show that when both reward sensitivity and attention problems and hyperactivity were self-reported, findings converged better, even though parent report of ADHD symptoms is generally considered as more valid (28). Note that, like attention problems and hyperactivity, reactive aggression was also rated by parents, suggesting that the effect on self-reported reward sensitivity on reactive regression is stronger than that on ADHD symptoms. Further, we also observed the hypothesized effect when exploring parent-rated fun-seeking rather than the total BAS score. Fun-seeking is potentially the most relevant for ADHD (32) and using the total BAS score might thus have underestimated the association with attention problems and hyperactivity. The literature is not fully consistent on the role of reward sensitivity in ADHD either. Gomez and Corr (32) reported in a cross-sectional study that ADHD symptoms were positively associated with reward sensitivity, and like here, more strongly for fun-seeking. In all, we conclude that although we identified a stable association between broad reward sensitivity and ADHD, the association may be, in fact, stronger for fun-seeking. It should be added here that the latter was also found for smoking, which fits with how fun-seeking has been linked to how smoking starts (33).

No evidence for the associations between reward sensitivity and autism spectrum problems and mood problems were found. As previously mentioned, the association between ASD and reward sensitivity had not been widely studied before. Therefore, our hypothesis that autism spectrum problems would be negatively associated with reward sensitivity was mainly based on prior neuroimaging and reaction time tasks research, which suggests a reduced or slower response to reward stimuli. It is unclear if these brain and cognitive responses tap into the same as reward sensitivity at the behavioral level. The neuroimaging and reaction time tasks were all cross-sectional, but the present cross-sectional associations (total BAS and subscales) were also fairly weak. Similar to autism spectrum problems, our results for mood problems were also not in line with our hypothesis of a negative association. Again, the negative associations are mostly found in relation to neuroimaging and reaction time task responses (34–36). In contrast, a recent meta-analysis including over 100 (cross-sectional) studies found a very small negative association between reward sensitivity and mood problems (6). However, the association was no longer significant when considering only self-rated mood problems, indicating that the present null finding fits within this pattern of small negative or no relations.

The main strength of our study is its prospective longitudinal design with a 14-year follow-up. TRAILS benefits from multiple measurement waves (here: five waves encompassing ages 13 to 26), and relatively high retention rates (12). Additionally, TRAILS is characterized by broad measurement, with the current study in particular, benefitting from the multiple psychopathology domains repeated over time. Our study bridges the current fragmentary knowledge on the association between reward sensitivity and psychopathology, in which research papers tend to focus on one type of psychopathology at a time, while we provided an overview of these associations across different domains of psychopathology. This work might be seen as a basis to further investigate the potential causal role of reward sensitivity in the onset and course of psychopathology. A potential limitation is that our findings do not directly translate to psychiatric disorders as we studied dimensional measures of psychopathology. Thus, the current study is predominantly useful for extending the available knowledge on the role of reward sensitivity in psychopathology rather than having immediate clinical impact. We nonetheless want to stress that given its long-lasting widespread relations with psychopathology, reward sensitivity could be a cross-diagnostic theme that may be probed during diagnostic assessment and potentially targeted in treatment. A second limitation is that we only used reward sensitivity to predict future change. Thus, there are two things to consider. First, even though reward sensitivity is thought to be stable over the lifespan, this has not been widely studied in longitudinal studies so far. For example, onset of symptoms of depression may, in turn, reduce reward sensitivity. We were unable to study change like these since we had no repeated measures of reward sensitivity. Second, we studied trajectories of homotypic continuity in the current paper, but psychopathology may change from one type of symptoms to another during development. For example, the currently identified stable link of reward sensitivity with ADHD and the increasing link with cannabis use may be partly driven by children with high ADHD symptoms who start using cannabis (37). Heterotypic continuity as a function of reward sensitivity was not addressed here, first, because the paper was already complex by including nine types of outcomes, and second, because larger samples are necessary for establishing such complex relations among these nine problem domains. We want to additionally note that the BAS scale is only one way to measure reward sensitivity. We would like to see confirmation of our longitudinal findings based on different reward sensitivity instruments.

In conclusion, our study showed that reward sensitivity has a long-lasting effect on the future course of psychopathology between adolescence and young adulthood. Thus, our work adds to the understanding of the role of reward sensitivity in psychopathology, providing an overview of the prospective associations across different psychopathology domains.

## Data Availability Statement

The data analyzed in this study is subject to the following licenses/restrictions: The datasets analyzed in this study are subject to the European Union's General Data Protection Regulation and are not publicly available. However, data can be requested by means of a publication plan. Requests to access these datasets should be directed to https://easy.dans.knaw.nl.

## Ethics Statement

The studies involving human participants were reviewed and approved by the Ethics Committee of the University Medical Center Groningen. Written informed consent to participate in this study was provided by the participants' legal guardian/next of kin.

## Author Contributions

RC analyzed the data and drafted the manuscript. RG and CH reviewed and edited the manuscript. All authors designed the study and approved the final manuscript.

## Funding

This research is part of the TRacking Adolescents' Individual Lives Survey (TRAILS) and the Comorbid Conditions of Attention-Deficit/Hyperactivity Disorder (CoCA). TRAILS has been financially supported by grants from the Netherlands Organization for Scientific Research NWO (Medical Research Council Program grant GB-MW 940-38-011; ZonMW Brainpower grant 100-001-004; ZonMw Risk Behaviour and Dependence grants 60-60600-97-118; ZonMw Culture and Health grant 261-98-710; Social Sciences Council Medium-Sized Investment grants GB-MaGW 480-01-006 and GB-MaGW 480-07-001; Social Sciences Council project grants GB-MaGW 452-04-314 and GB-MaGW 452-06-004; NWO Large-Sized Investment grant 175.010.2003.005; NWO Longitudinal Survey and Panel Funding 481-08-013 and 481-11-001; NWO Vici 016.130.002 and 453-16-007/2735; NWO Gravitation 024.001.003), the Dutch Ministry of Justice (WODC), the European Science Foundation (EuroSTRESS project FP-006), the European Research Council (ERC-2017-STG-757364 and ERC-CoG-2015-681466), Biobanking and Biomolecular Resources Research Infrastructure BBMRI-NL (CP 32), the Gratama Foundation, the Jan Dekker Foundation, the participating universities, and Accare Centre for Child and Adolescent Psychiatry. CoCA has received funding from the European Union's Horizon 2020 Research and Innovation Programme under grant agreement no. 667302.

## Conflict of Interest

The authors declare that the research was conducted in the absence of any commercial or financial relationships that could be construed as a potential conflict of interest.

## Publisher's Note

All claims expressed in this article are solely those of the authors and do not necessarily represent those of their affiliated organizations, or those of the publisher, the editors and the reviewers. Any product that may be evaluated in this article, or claim that may be made by its manufacturer, is not guaranteed or endorsed by the publisher.

## Footnotes

^1^Please note that this is a deviation from our preregistration. We previously hypothesized that reward sensitivity would be negatively associated with anxiety problems based on the strong association between anxiety and depression. However, we had missed highly relevant literature supporting a positive association. Further details are provided in Supplementary Data Sheet S1.

^2^Please note that this is a deviation from our preregistration. Correlation analyses using the BAS subscales were added to explore whether the different aspects of reward sensitivity would be differently associated with the psychopathology domains. Further details are provided in Supplementary Data Sheet S1.

^3^Please note that this is a deviation from our preregistration. These sensitivity analyses were added to explore whether our results were influenced by psychotropic medication use, which we had accidentally not considered upon pre-registration. Further details are provided in Supplementary Data Sheet S1.
