# Supplementary material for: Reward Sensitivity at Age 13 Predicts the Future Course of Psychopathology Symptoms
Source: Front Psychiatry. 2022 Mar 11;13:818047. doi: 10.3389/fpsyt.2022.818047 (PMC8962629; doi:10.3389/fpsyt.2022.818047)
Supplement: Supplementary file 1 [file Data_Sheet_1.docx]

Supplementary Material

Reward sensitivity at age 13 predicts the future course of psychopathology symptoms

Raniere Dener Cardoso Melo^1^, Robin N. Groen^1^, Catharina A. Hartman^1^

^1^Interdisciplinary Center Psychopathology and Emotion Regulation (ICPE), Department of Psychiatry, University Medical Center Groningen, University of Groningen, Groningen, Netherlands

*** Correspondence:**
Raniere Dener Cardoso Melo
r.d.cardoso.melo@umcg.nl

# Supplement S1 - Preregistration and deviations

The aims, hypotheses, and analyses of this study were preregistered prior to analyzing the data. The preregistration form and code can be downloaded via <https://osf.io/47qwk>. TRAILS data can be requested by means of a publication plan via DANS EASY (https://easy.dans.knaw.nl).

Deviations from the hypotheses and analyses described in the preregistration are explained here. The first deviation is regarding the hypothesis on the association between reward sensitivity and anxiety problems. The evidence on the relationship between reward sensitivity and anxiety disorders is somewhat mixed. However, after preregistering, we noticed that we had missed some highly relevant literature supporting a positive association instead of a negative one. Initially, we hypothesized that reward sensitivity would be negatively associated with anxiety problems based on the strong association between anxiety and depression. However, Gray and McNaughton suggests that anxiety can be explained by an approach-avoidance conflict, in which there is the activation of both reward and punishment systems that typically happens in highly novel, ambiguous, and unpredictable contexts, resulting in an approach-avoidance conflict (1). Further, Barker and colleagues have reviewed findings from clinical and cognitive neuroscience studies that also support an increased reward and punishment sensitivity in anxiety (2). Therefore, we hypothesized a positive association in which we would see more worsening over time in anxiety problems for individuals with higher reward sensitivity levels than individuals with lower reward sensitivity. The second deviation is regarding the correlation analysis using the BAS subscales. Previous research has pointed out the relevance of considering the different aspects of reward sensitivity when analyzing its relationship with psychopathology (3). However, this literature is complex, and we, therefore, decided that our main analyses should be with the total BAS scale. Following the preregistration, however, we added correlation analyses using the BAS subscales to explore whether the different subscales would be differently associated with the psychopathology domains and did post-hoc model fitting based on these results as described in the paper. Finally, the third deviation is regarding extra sensitivity analyses on psychotropic medication use. Psychopathological problems are directly influenced by psychotropic medication use. We realized after preregistration that medication use could have influenced the estimation of the effect of reward sensitivity on psychopathology. Therefore, we adjusted our analyses for relevant psychotropic medication for each of the psychopathology domains. We adjusted for psychostimulant use for attention problems and hyperactivity, smoking, alcohol use, and cannabis use. For mood and anxiety problems, we adjusted for antidepressants and sedative use. Finally, for reactive and proactive aggression problems, we adjusted for antipsychotics use, the latter given that these are sometimes prescribed for severe aggression.

# Supplement S2 - Missing data and imputation procedure

We computed mean scores for reward sensitivity. For each of the psychopathology problem domain scales, we computed mean scores at each wave. Participants who had more than 50% of items missing from a scale were coded as missing. For participants with less than 50% of the items per scale missing, we used the corrected item mean imputation (CIM) method to estimate the scale mean score. CIM imputation (4,5) replaces item non-response with the sample mean corrected for a weighting factor, reflecting the ratio of the respondent’s score (on the items that were filled in) and the average score in the sample. This procedure does not apply to substance use variables as they were not measured using multiple items. For substance use variables, we used only the data available for each participant.

Parent-rated scores on attention problems and hyperactivity and reactive aggression problems were not collected at waves T4 and T6. Therefore, we imputed them. For that, a linear mixed model was fitted. Parent-rated scores at waves T1, T2, T3, and T5 and self-rated scores at waves T1, T2, T3, T4, T5, and T6 were used as outcome variables. The follow-up time since enrollment (measured in years on a continuous scale) and informant type (parent or self) were the predictors in the model. Subject-level intercepts and slopes from the model were extracted. The residual variance was used to draw a random residual value from a normal distribution with a mean zero and this variance. These parameters were used to build participant-specific regression equations, as shown below, to estimate individual parent ratings for each participant.

Yj = β0j + β1j (t) + β2j (I)+ е, where Yj denotes estimated score for a jth subject, β0j is the intercept, β1j is the slope for follow-up time, t is the follow-up time in years, β2j is the slope for informant type (parent-rated coded as one and self-rated as zero), and e is the randomly drawn residual for a jth subject.

The equation was used 50 times per participant, yielding 50 estimates of parent ratings. These scores were subsequently pooled to a single imputed value for each participant. To ensure that the imputed score was valid, we imputed only for those study participants who had, at most, one missing value out of the four parent-ratings of waves T1, T2, T3, and T5. In addition, imputed values that were slightly below zero or above two (maximum mean score) were constrained to be zero and two, respectively.

Parent-rated scores on autism spectrum problems were not collected at wave T5 for the general population cohort. Therefore, we imputed these using the information available from the other waves and the clinical cohort data at wave T5. A similar procedure as the one described above was used. However, since we used parent ratings of autism spectrum problems, we used only parent-rated scores at waves T1, T2, T3, T4, T5 (from the clinical cohort), and T6 as outcomes variables and follow-up time since enrollment as the predictor in the model. Therefore, the participant-specific equations did not include the slope term for informant type: Yj = β0j + β1j (t) + е. We used the same criteria and imputed only for participants who had, at most, one missing value out of the five measures for autism spectrum problems.

# Supplement S3 - Self-rated substance use problems

Smoking was assessed at waves T2, T3, T4, T5, and T6. We measured the self-rated frequency of cigarette smoking in the past month. At T2 and T3, response categories ranged from 0= never smoked to 7= smoked > 20 cigarettes a day in the previous month. At T4, T5, and T6, response categories ranged from 1= “did not smoke in the previous month” to 8= “smoked >30 cigarettes a day in the previous month.” To have comparable response categories across all waves, we combined categories 0 and 1 into one category. Similarly, we combined categories 7 and 8. Therefore, smoking was scored on a 7-point scale (coded as 0 ‘no cigarettes’/ 6 ‘more than 20 cigarettes’).

Alcohol use was assessed at waves T2, T3, T4, T5, and T6. We calculated a mean score reflecting the average number of alcoholic beverages consumed during a regular day. Individuals were asked how many days a week and weekend they had something to drink and how many glasses they would have on a regular weekday and weekend day. Based on the number of drinking days and the number of drinks per day, we estimated the average number of alcoholic beverages consumed during a day. To deal with outliers, we trimmed extreme values to a maximum of five standard deviations. Therefore, alcohol use was scored on a scale that ranged from zero to 20 beverages/day.

Cannabis use was assessed at waves T2, T3, T4, T5, and T6. We measured the self-rated frequency of monthly cannabis use. From T2 until T5, individuals were asked how many times they had used cannabis in the past four weeks. At T6, individuals were asked how many times they had used cannabis in the last three months. We recoded the response categories at T6 to represent the monthly use, thus having comparable response categories across all waves. Therefore, cannabis use was scored on a scale that ranged from zero to 40 times.

Table S1. Multilevel growth curve model estimates for all psychopathology domains when using only comparable items over time, without overlapping items between reactive and proactive aggression, and using self-rated attention problems and hyperactivity.

|  | **Comparable items** | | | | | | **Aggression without overlap** | **Self-rated** |
| --- | --- | --- | --- | --- | --- | --- | --- | --- |
|  | **Attention and hyperactivity** | **Autism spectrum** | **Reactive aggression** | **Proactive aggression** | **Mood** | **Anxiety** | **Proactive**  **aggression** | **Attention and hyperactivity** |
|  | **Est (SE)** | **Est (SE)** | **Est (SE)** | **Est (SE)** | **Est (SE)** | **Est (SE)** | **Est (SE)** | **Est (SE)** |
| Fixed Effects |  |  |  |  |  |  |  |  |
| (Intercept) | -0.14 (0.18) | -0.01 (0.12) | **0.31 (0.12)*** | **-0.24 (0.08)*** | **0.70 (0.19)**** | 0.32 (0.26) | **-0.24 (0.08)*** | 0.31(0.24) |
| Follow-up time | **-0.45 (0.03)**** | **-0.14 (0.02)**** | **-0.65 (0.02)**** | **-0.24 (0.01)**** | **0.38 (0.04)**** | **0.11 (0.05)*** | **-0.24 (0.01)**** | **-1.20 (0.04)**** |
| Reward sensitivity | 0.21 (0.13) | 0.00 (0.09) | **0.26 (0.10)*** | **0.21 (0.06)*** | 0.26 (0.14) | **0.42 (0.20)*** | **0.21 (0.06)*** | **0.57 (0.19)*** |
| Reward sensitivity*Time | 0.03 (0.03) | 0.01 (0.02) | -0.00 (0.02) | 0.01 (0.01) | 0.02 (0.04) | 0.01 (0.05) | 0.01 (0.01) | 0.03 (0.04) |
| Baseline psychopathology | **-1.84 (0.14)**** | **-1.15 (0.09)**** | **-1.52 (0.10)**** | **-1.02 (0.07)**** | **-2.05 (0.14)**** | **-3.76 (0.20)**** | **-1.02 (0.07)**** | **-2.79 (0.19)**** |
| Baseline psychopathology *Time | **-0.70 (0.03)**** | **-0.44 (0.02)**** | **-0.73 (0.02)**** | **-0.48 (0.01)**** | **-0.57 (0.04)**** | **-0.88 (0.05)**** | **-0.48 (0.01)**** | **-1.22 (0.04)**** |
| Baseline age | **-0.49 (0.12)**** | **-0.21 (0.08)*** | **-0.38 (0.08)**** | **-0.20 (0.05)**** | 0.08 (0.13) | 0.02 (0.17) | **-0.20 (0.05)**** | **-0.71 (0.16)**** |
| Sex | 0.33 (0.24) | 0.04 (0.16) | -0.16 (0.15) | **0.81 (0.10)**** | **-2.27 (0.25)**** | **-3.19 (0.35)**** | **0.81 (0.10)**** | -0.01 (0.31) |
| SES | -0.19 (0.13) | **-0.23 (0.09)*** | **-0.22 (0.08)*** | **-0.16 (0.06)*** | -0.17 (0.14) | -0.13 (0.18) | **-0.16 (0.06)*** | **-0.35 (0.17)*** |
| IQ | 0.10 (0.13) | **-0.29 (0.09)*** | **-0.19 (0.08)*** | **-0.18 (0.06)*** | 0.14 (0.14) | **-0.59 (0.18)*** | **-0.18 (0.06)*** | -0.05 (0.17) |
| Random effects |  |  |  |  |  |  |  |  |
| Variance | 1.24 | 0.59 | 0.30 | 0.12 | 1.57 | 2.64 | 0.12 | 1.75 |
| Residual variance | 62.90 | 29.45 | 31.71 | 15.54 | 79.39 | 149.37 | 15.54 | 130.01 |
| ICC | 0.50 | 0.49 | 0.32 | 0.27 | 0.48 | 0.45 | 0.27 | 0.38 |

Est, estimate; SE, standard error; values in bold are statistically significant; *****, p < 0.05; ******, p < 0.001.

Table S2. Multilevel growth curve model estimates for all psychopathology domains adjusted for medication use.

|  | **Attention and hyperactivity** | **Reactive aggression** | **Proactive Aggression** | **Mood** | **Anxiety** | **Smoking** | **Alcohol** | **Cannabis** |
| --- | --- | --- | --- | --- | --- | --- | --- | --- |
|  | **Est (SE)** | **Est (SE)** | **Est (SE)** | **Est (SE)** | **Est (SE)** | **Est (SE)** | **Est (SE)** | **Est (SE)** |
| Fixed Effects |  |  |  |  |  |  |  |  |
| (Intercept) | **-0.51 (0.18)*** | 0.06 (0.13) | **-0.91 (0.08)**** | **0.54 (0.20)*** | -0.17 (0.23) | **4.56 (0.42)**** | **0.47 (0.11)**** | -0.13 (0.21) |
| Follow-up time | **-0.74 (0.03)**** | **-0.38 (0.02)**** | **-0.53 (0.01)**** | **0.39 (0.04)**** | **0.63 (0.05)**** | **1.91 (0.09)**** | **0.56 (0.02)**** | **0.55 (0.05)**** |
| Reward sensitivity | 0.18 (0.13) | 0.19 (0.10) | **0.21 (0.07)*** | 0.21 (0.15) | **0.34 (0.17)*** | 0.03 (0.31) | 0.02 (0.08) | 0.12 (0.16) |
| Reward sensitivity*Time | 0.04 (0.03) | 0.01 (0.02) | -0.00 (0.01) | 0.01 (0.04) | 0.01 (0.05) | **0.20 (0.09)*** | **0.06 (0.02)*** | **0.11 (0.05)*** |
| Baseline psychopathology | **-2.81 (0.15)**** | **-1.55 (0.10)**** | **-1.58 (0.07)**** | **-2.28 (0.15)**** | **-3.42 (0.17)**** | **-1.23 (0.34)**** | -0.02 (0.09) | **-0.80 (0.21)**** |
| Baseline psychopathology *Time | **-1.02 (0.03)**** | **-0.68 (0.02)**** | **-0.63 (0.01)**** | **-0.58 (0.04)**** | **-0.76 (0.05)**** | **-0.92 (0.10)**** | **-0.14 (0.02)**** | **-0.13 (0.06)*** |
| Baseline age | **-0.26 (0.12)*** | **-0.32 (0.08)**** | **-0.20 (0.04)**** | 0.13 (0.13) | 0.10 (0.15) | 0.10 (0.29) | **0.29 (0.07)**** | **0.37 (0.15)*** |
| Sex | 0.28 (0.23) | **-0.33 (0.17)*** | **1.00 (0.09)**** | **-2.37 (0.26)**** | **-2.78 (0.31)**** | -0.79 (0.57) | **1.47 (0.14)**** | **1.43 (0.29)**** |
| SES | **-0.30 (0.12)*** | **-0.23 (0.09)*** | **-0.16 (0.05)**** | -0.12 (0.14) | -0.11 (0.16) | **-1.23 (0.30)**** | **0.16 (0.08)*** | 0.12 (0.16) |
| IQ | -0.07 (0.12) | **-0.20 (0.09)*** | **-0.13 (0.05)**** | 0.11 (0.14) | **-0.47 (0.16)*** | **-0.96 (0.30)*** | 0.05 (0.08) | 0.01 (0.16) |
| Medication use | **4.77 (0.40)**** | **3.27 (0.51)**** | 0.42 (0.32) | **4.62 (0.63)**** | **2.73 (0.70)**** | **3.25 (0.90)**** | -0.10 (0.23) | -0.63 (0.46) |
| Random effects |  |  |  |  |  |  |  |  |
| Variance | 1.07 | 0.63 | 0.01 | 1.58 | 3.14 | 10.79 | 0.43 | 3.17 |
| Residual variance | 59.63 | 31.86 | 16.11 | 80.35 | 95.79 | 333.55 | 22.97 | 85.92 |
| ICC | 0.45 | 0.47 | 0.03 | 0.48 | 0.61 | 0.61 | 0.43 | 0.64 |

Est, estimate; SE, standard error; values in bold are statistically significant; *****, p < 0.05; ******, p < 0.001.

Table S3. Multilevel growth curve model estimates for all psychopathology domains when using the BAS subscale strongest correlated with each domain.

|  | **Attention and hyperactivity** | | **Autism spectrum** | **Reactive aggression** | **Proactive Aggression** | **Mood** | **Anxiety** | **Smoking** | **Alcohol** | **Cannabis** |
| --- | --- | --- | --- | --- | --- | --- | --- | --- | --- | --- |
|  | **Est (SE)** | | **Est (SE)** | **Est (SE)** | **Est (SE)** | **Est (SE)** | **Est (SE)** | **Est (SE)** | **Est (SE)** | **Est (SE)** |
|  | **Parent-rated** | **Self-rated** |  |  |  |  |  |  |  |  |
| Fixed Effects |  |  |  |  |  |  |  |  |  |  |
| (Intercept) | -0.17 (0.18) | 0.30 (0.24) | -0.05 (0.13) | 0.15 (0.13) | **-0.77 (0.08)**** | **0.72 (0.19)**** | -0.08 (0.22) | **4.70 (0.40)**** | **0.51 (0.10)**** | -0.09 (0.21) |
| Follow-up time | **-0.75 (0.03)**** | **1.20 (0.04)**** | -0.04 (0.02) | **-0.38 (0.02)**** | **-0.54 (0.01)**** | **0.38 (0.04)**** | **0.62 (0.05)**** | **1.95 (0.09)**** | **0.56 (0.02)**** | **0.54 (0.05)**** |
| Reward sensitivity | **0.39 (0.13)*** | **0.55 (0.19)*** | 0.03 (0.09) | 0.18 (0.10) | **0.22 (0.07)*** | 0.27 (0.15) | 0.23 (0.16) | **0.88 (0.30)*** | 0.04 (0.08) | **0.48 (0.15)*** |
| Reward sensitivity*Time | 0.04 (0.03) | 0.04 (0.04) | 0.02 (0.02) | -0.01 (0.02) | -0.00 (0.01) | -0.02 (0.04) | 0.07 (0.05) | **0.22 (0.09)*** | **0.06 (0.02)*** | 0.08 (0.05) |
| Baseline psychopathology | **-2.34 (0.14)**** | **-2.79 (0.19)**** | **-1.18 (0.10)**** | **-1.54 (0.10)**** | **-1.48 (0.07)**** | **-2.13 (0.15)**** | **-3.33 (0.16)**** | **-1.24 (0.32)**** | 0.00 (0.08) | **-0.46 (0.17)*** |
| Baseline psychopathology*Time | **-1.00 (0.03)**** | **-1.22 (0.04)**** | **-0.39 (0.02)**** | **-0.64 (0.02)**** | **-0.64 (0.01)**** | **-0.60 (0.04)**** | **-0.80 (0.05)**** | **-0.98 (0.10)**** | **-0.14 (0.02)**** | **-0.19 (0.06)*** |
| Baseline age | **-0.48 (0.12)**** | **-0.73 (0.16)**** | **-0.30 (0.09)*** | **-0.38 (0.08)**** | **-0.22 (0.04)**** | 0.06 (0.13) | 0.09 (0.15) | -0.16 (0.27) | **0.28 (0.07)**** | **0.35 (0.14)*** |
| Sex | **0.58 (0.23)*** | 0.00 (0.31) | 0.22 (0.17) | **-0.34 (0.17)*** | **1.02 (0.09)**** | **-2.28 (0.26)**** | **-2.71 (0.29)**** | -0.52 (0.54) | **1.45 (0.14)**** | **1.32 (0.28)**** |
| SES | **-0.27 (0.12)*** | **-0.34 (0.17)*** | **-0.27 (0.09)*** | **-0.23 (0.09)*** | **-0.19 (0.05)**** | -0.18 (0.14) | -0.14 (0.16) | **-1.19 (0.29)**** | 0.14 (0.07) | 0.11 (0.15) |
| IQ | -0.05 (0.13) | -0.07 (0.17) | **-0.24 (0.09)*** | **-0.19 (0.09)*** | **-0.20 (0.05)**** | 0.12 (0.14) | **-0.47 (0.16)*** | **-1.02 (0.29)*** | 0.02 (0.07) | 0.04 (0.15) |
| Random effects |  |  |  |  |  |  |  |  |  |  |
| Variance | 1.03 | 1.75 | 0.71 | 0.60 | 0.01 | 1.63 | 3.22 | 11.36 | 0.44 | 3.01 |
| Residual variance | 62.00 | 130.05 | 31.59 | 32.79 | 17.06 | 80.27 | 96.14 | 336.88 | 23.37 | 88.34 |
| ICC | 0.46 | 0.38 | 0.52 | 0.48 | 0.02 | 0.48 | 0.60 | 0.61 | 0.46 | 0.61 |

Est, estimate; SE, standard error; values in bold are statistically significant; *****, p < 0.05; ******, p < 0.001.

**Figure S1. Associations between the highest correlated BAS subscale and psychopathology domains with similar effects compared to total BAS effects.** The figures display the effect of reward sensitivity on psychopathology based on its main effect and in interaction with time. Here, the x-axes show follow-up time in years, whereas the different lines show reward sensitivity levels at 2 SD above and below the mean. The y-axes represent the predicted outcome with all covariates at mean levels and sex coded as 0 (males). The y-axes are only partly shown for better visualization of the findings.


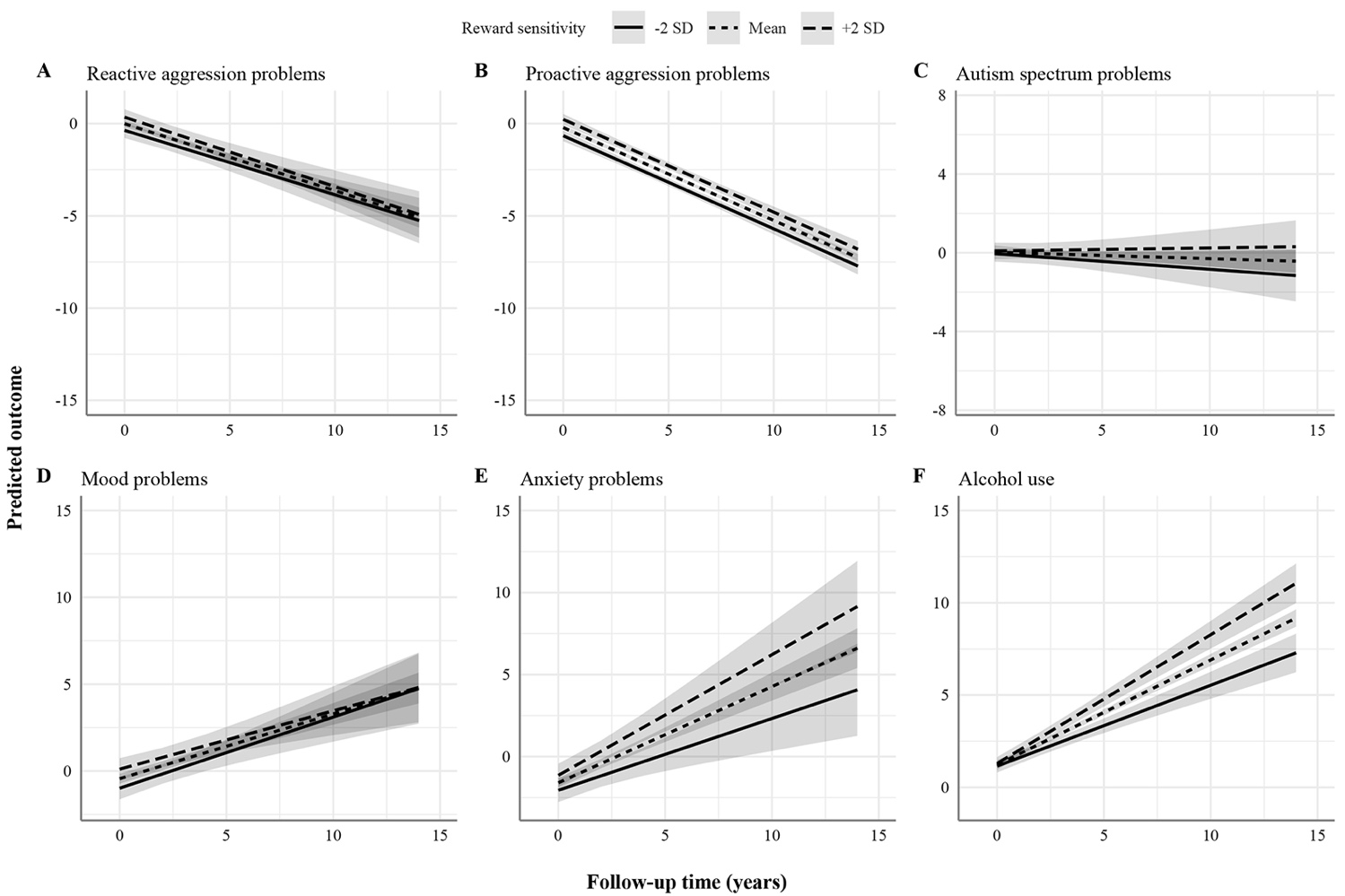


**Figure S2. Associations between the highest correlated BAS subscale and psychopathology domains with bigger effects compared to BAS total effects.** The figures display the effect of reward sensitivity on psychopathology based on its main effect and in interaction with time. Here, the x-axes show follow-up time in years, whereas the different lines show reward sensitivity levels at 2 SD above and below the mean. The y-axes represent the predicted outcome with all covariates at mean levels and sex coded as 0 (males). The y-axes are only partly shown for better visualization of the findings.


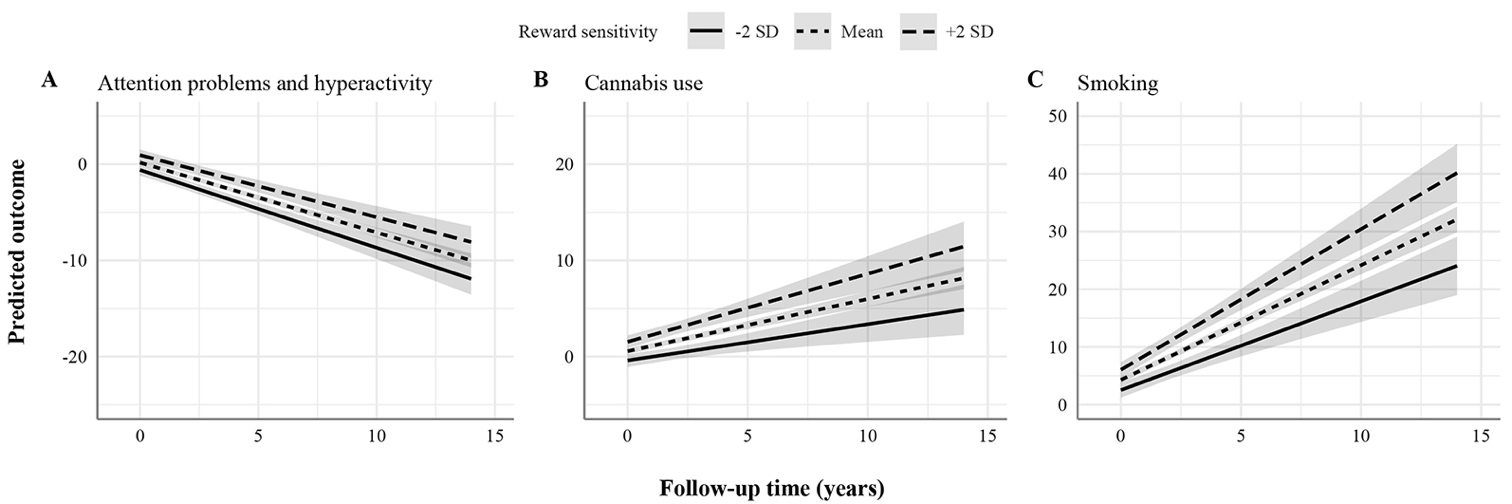


**Figure S3. Association between reward sensitivity and self-rated attention problems and hyperactivity.** The figure displays the effect of reward sensitivity on self-rated attention problems and hyperactivity based on its main effect and in interaction with time. Here, the x-axis shows follow-up time in years, whereas the different lines show reward sensitivity levels at 2 SD above and below the mean. The y-axis represents the predicted outcome with all covariates at mean levels and sex coded as 0 (males). The y-axis is only partly shown for better visualization of the findings.


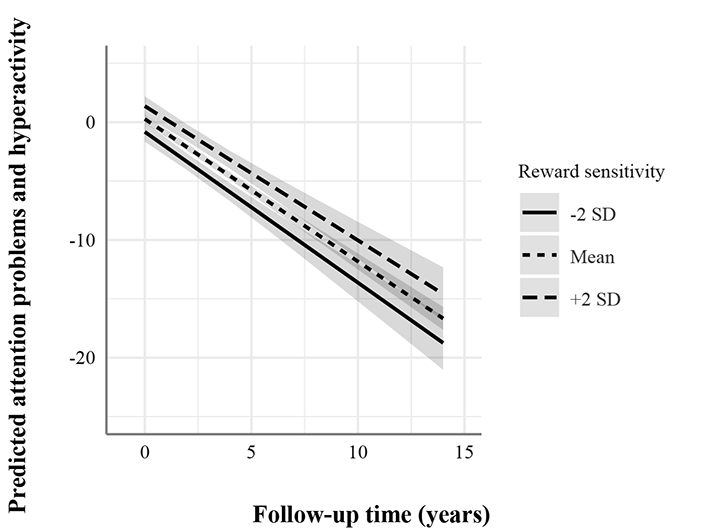


# References

(1) Gray JA, McNaughton N. The Neuropsychology of Anxiety. Oxford: Oxford University Press; 2003.

(2) Barker TV, Buzzell GA, Fox NA. Approach, avoidance, and the detection of conflict in the development of behavioral inhibition. New ideas in psychology 2019 Apr;53:2-12.

(3) Zald DH, Treadway MT. Reward Processing, Neuroeconomics, and Psychopathology. Annual Review of Clinical Psychology 2017;13(1):471-495.

(4) Mark Huisman. Item Nonresponse: Occurence, Causes, and Imputation of Missing Answers to Test Items; 1999.

(5) Huisman M. Imputation of missing item responses: Some simple techniques. Quality & Quantity 2000 Nov;34(4):331-351.

stylefix
